# Examination of the Relationship between Psychosocial Mediators and Intervention Effects in It's Your Game: An Effective HIV/STI/Pregnancy Prevention Intervention for Middle School Students

**DOI:** 10.1155/2012/298494

**Published:** 2012-06-28

**Authors:** Elizabeth Baumler, Jill Glassman, Susan Tortolero, Christine Markham, Ross Shegog, Melissa Peskin, Robert Addy, Heather Franks

**Affiliations:** ^1^Center for Health Promotion and Prevention Research, University of Texas Health Science Center at Houston, 7000 Fannin, Suite 2200, Houston, TX 77030, USA; ^2^ETR Associates, Scotts Valley, CA 95066, USA

## Abstract

A set of mediation analyses were carried out in this study using data from It's Your Game. . .Keep It Real (IYG), a successful HIV/STI/pregnancy prevention program. The IYG study evaluated a skill and normbased. HIV/STI/pregnancy prevention program that was implemented from 2004 to 2007 among 907 urban low-income middle school youth in Houston, TX, USA. Analyses were carried out to investigate the degree to which a set of proposed psychosocial measures of behavioral knowledge, perceived self-efficacy, behavioral, and normative beliefs, and perceived risky situations, all targeted by the intervention, mediated the intervention's effectiveness in reducing initiation of sex. The mediation process was assessed by examining the significance and size of the estimated effects from the mediating pathways. The findings from this study provide evidence that the majority of the psychosocial mediators targeted by the IYG intervention are indeed related to the desired behavior and provide evidence that the conceptual theory underlying the targeted psychosocial mediators in the intervention is appropriate. Two of the psychosocial mediators significantly mediated the intervention effect, knowledge of STI signs and symptoms and refusal self-efficacy. This study suggests that the underlying causal mechanisms of action of these interventions are complex and warrant further analyses.

## 1. Introduction 

The majority of teen HIV/STI/pregnancy prevention programs are theory-based, targeting psychosocial variables to produce changes in sexual risk-taking behaviors. Guided by established psychosocial theories such as social cognitive theory [1] and social influence theory [2, 3], these interventions seek to reduce sexual risk-taking behaviors, such as sexual initiation and frequency of sex, first by impacting psychosocial mediating factors, such as attitudes and self-efficacy regarding those behaviors [4–6]. Researchers in the field recognize a critical need for further examination of psychosocial mediating factors to gain insight into the mechanisms of action influencing behavior change for these interventions [4–6] because there are little data available on which psychosocial variables provide the actual mediating causal mechanisms through which HIV/STI/pregnancy prevention interventions change sexual risk-taking behaviors for adolescent populations. In this study a set of mediation analyses was carried out using data from It's Your Game…Keep It Real (IYG), a successful HIV, STI, and pregnancy prevention program [7, 8] to investigate the degree to which the psychosocial outcomes mediated the intervention effect.

Adolescents are engaging in sexual risk-taking behaviors at an earlier age, often before they are developmentally ready to deal with potential outcomes. National data illustrate the need to implement intervention programs as early as possible: 11% of 6th graders, 15% of 7th graders, 18% of 8th graders, and 33% of 9th graders have reported lifetime sexual activity [9, 10]. This rapid acquisition of sexual behavior clearly demonstrates that early comprehensive sex education such as IYG is imperative. There is a critical need to develop effective HIV, STD, and pregnancy prevention programs for students in middle schools, to help delay or mitigate the consequences of early sexual activity.

IYG was developed using a systematic design process, Intervention Mapping [11], and was grounded in social cognitive theories [1, 12]. Intervention Mapping is a detailed process that provides researchers with a systematic method for decision making in each phase of developing an intervention to influence changes in behavior. Intervention Mapping uses theory, empirical evidence, and participant involvement to review program objectives, theory-based methods, strategies, plans for program adoption and implementation, and refinement of evaluation instruments. Social cognitive theory emphasizes interactions between personal (e.g., behavioral knowledge, perceived self-efficacy), environmental (e.g., exposure to risky situations), and behavioral influences (e.g., dating relationships) [1]. The theory of planned behavior emphasizes interactions between behavioral beliefs, normative beliefs (e.g., the beliefs of influential others, such as peers or parents), intentions, and behavior [12]. IYG activities were designed to positively impact behavioral knowledge, self-efficacy, behavioral and normative beliefs, intentions, and environmental factors related to healthy dating relationships and delayed sexual initiation. 

A mediation analysis involves performing a set of regression analyses to obtain estimates of (1) the strength of the relationship between an independent variable *X* and a hypothesized mediator variable *M*, and (2) the strength of the relationship between the mediator variable *M* and the dependent variable *Y*, adjusting for the effects of *X*; combining these estimates provides a measure of the strength of the mediated or indirect effect of *X* on *Y* through mediator variable *M*. A key assumption of mediation hypotheses is that there is a causal chain of events in which *X* occurs and produces a subsequent change in *M*, which in turn causes a subsequent change in *Y* [13, 14]. Of interest is the amount of change in *Y* caused by *X* indirectly transmitting change through *M*, that is, the indirect or mediated effect. This indirect effect may be whole or partial, accounting for a proportion of the total effect of *X* on *Y*. 

## 2. Methods

### 2.1. Study Design

The It's Your Game (IYG) study evaluated a skill and norm-based HIV, STI, and pregnancy prevention program that was implemented from 2004 to 2007 among urban low-income middle school youth in Houston, TX, USA. Ten predominately African American and Hispanic middle schools were randomly assigned to either the intervention or comparison condition (five schools to each) using a multiattribute randomization protocol, taking into account the size and racial/ethnic composition of the student body (African American and Hispanic) and the geographic location of the school [15]. Participants at the intervention schools received the IYG intervention, delivered in the 7th and 8th grades; those in the comparison condition received their regular health classes, which varied by school. The IYG intervention was composed of twelve 7th-grade and twelve 8th-grade 45-minute lessons. The IYG intervention was delivered once a day over a two-three week period or once or twice a week over a 6–12 week period, depending on if the school had a blocked schedule or not. The cohort of students (*n* = 907, 588 control, 349 treatment) was followed over a period of two years. Over the study period students were surveyed at baseline, 6, 18, and 24 months using laptop computers via an audio-computer assisted self-interview (ACASI) [16]. Upon completion of the study, almost 30% of the students in the comparison group had initiated sex by ninth grade, compared with 23% of students who received IYG, a difference that was statistically significant. For further details about the study design and main outcome evaluation, please see Tortolero et al [7].

### 2.2. Measures

The primary behavioral outcome for IYG was delay of sexual initiation for those students who reported no lifetime sexual activity at baseline. This was used as the primary behavior (*Y*) in the mediation models. Table 1 shows the psychosocial mediators that were used for this analysis. The mediators used in this study were taken from the IYG main outcome study. The IYG intervention was grounded in theory and specifically developed to target all of the psychosocial mediators presented in this study.

The analyses include some constructs that may not initially be expected to be associated with delayed initiation (e.g., *condom knowledge*, *self-efficacy to use condoms*, *perceived norms about condom use*). These mediators were included because condom skills training is a recommended element of sexual health education at the middle school level [17]. It is important to assess whether inclusion of this type of information adversely impacts delay of sexual debut. 

### 2.3. Intervention

IYG comprised 24-, 50-minute lessons, with twelve lessons in 7th grade and twelve lessons in 8th grade. It integrated group-based classroom activities with individual journaling and computer-based activities. Computer activities included a virtual world interface, educational activities (e.g., interactive skills-training exercises, peer role model videos) tailored by gender and sexual experience, and “real world”-style teen serials with on-line student feedback. IYG also included six homeworks to facilitate parent-child communication.

Specific topics covered in the IYG intervention included characteristics of healthy friendships, setting personal limits and practicing refusal skills in a general context (e.g., regarding alcohol and drug use, skipping school, cheating), information about puberty, reproduction, and STIs, and setting personal limits and practicing refusal skills related to sexual behavior, characteristics of healthy dating relationships, the importance of HIV, STI, and pregnancy testing if a person is sexually active, and skills training regarding condom and contraceptive use [8].

### 2.4. Mediation Model Framework

**Figure 1:**
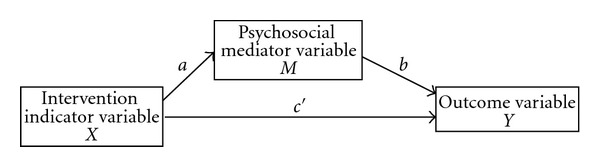
Single mediator process.

Figure 1 shows a path diagram for the simplest single mediator model, and (1)–(3) show the corresponding regression equations for performing a mediation analysis of the indirect effect of *X* on *Y* through *M*. For the present study, *X* is the intervention indicator variable, taking a value of 0 for those students in the control arm and a value of 1 for those in the intervention arm of the IYG study. The outcome of interest is represented by the variable *Y* (i.e., initiation of sexual intercourse), measured at the 24-month followup. The hypothesized psychosocial mediators are represented by the variable *M* (e.g., refusal self-efficacy) and were also measured at the 24-month followup:

(1)Y=i1+cX+e1,(2)Y=i2+c′X+bM+e2,(3)M=i3+aX+e3.

In these equations, *c *is the total effect of *X* on *Y*, and this can be decomposed into a direct effect, *c*′, and an indirect effect, *ab*, which is carried through the mediator *M*. The *e* terms represent the random error component of each model. In the case of single-level ordinary least squares (OLS) regression with an underlying continuous outcome variable, *ab* is equal to *c* − *c*′, which is the difference between the total effect and the direct effect of *X* on *Y* [13]. For multilevel regression models and regression models for categorical outcomes (e.g., logistic regression for a dichotomous outcome), *ab* does not equal *c* − *c*′, although the difference is generally not large, especially for large samples [13]. In logistic regression (single or multilevel), where the scales of the various mediation regression equations are different, standardization of estimates minimizes the difference between *ab* and *c* − *c*′ and allows for estimation of standard errors of the mediated effect. In this study *ab* rather than *c* − *c*′ was used to estimate mediated (indirect) effects [13]. 

### 2.5. Analysis Approach

The mediation process was assessed by examining the significance and size of the *a* and *b* effect estimates using the Wald tests *and *by examining the significance and size of the *ab* effect estimate using the Sobel test [13, 18, 19]. Although it has been argued that significance of the *a* and *b* effects is not necessary steps prior to conducting a test of whether *ab* is significant and therefore whether a variable is a significant mediator [19], these are nevertheless important to assess given the specific insight they provide into the “action” and “conceptual” theories underlying *how* an intervention *X* may produce change in an outcome *Y* [13]. Equation (2) provides a way for assessing the action theory of an intervention, which suggests how the intervention should be designed to affect a given psychosocial variable. Equation (3) provides a way of obtaining evidence about the conceptual theory underlying an intervention, which is the psychosocial theory (e.g., social cognitive theory) upon which the choice of targeted psychosocial mediators was based. Thus, significance of *b* and not *a* suggests either that the mediator being targeted is correct but that the intervention needs to be strengthened in its strategies for influencing the mediator or that the mediator needs to be measured more accurately. Significance of *a* but not *b* suggests that although the intervention is successful in influencing the targeted mediator, the psychosocial theory dictating which mediator to target needs some refinement [13]. Therefore this study assesses both the direction and significance of *a* and *b* individually as well as the significance of the mediated effect *ab*. 

### 2.6. Test for Significance of Mediated Effects

One of the most common methods for estimating the standard error (SE) of a mediated effect is to use the formula first derived by Sobel based on the multivariate delta method [13, 18, 19]:

(4)sab=a2sb2+b2sa2.

It can be used in the case of logistic regression with a dichotomous outcome, such is the case with this study, as long as regression parameter estimates and their accompanying SEs are first standardized [13]. This tests for the significance of a mediated effect (Sobel test), in which the estimate *ab* is divided by its Sobel SE and compared to a critical value from a standard normal distribution and was used to assess mediation effects in the present study [18, 19]. 

The sample was restricted to those students reporting no sexual experience at the 7th-grade baseline measure (*n* = 817), in order to look at the main behavioral outcome of initiation of sexual intercourse in 9th grade. The psychosocial mediators were also analyzed on this same subset in order to carry out the mediational analyses. The IYG study design consisted of students nested within schools. In keeping with the analysis of the original study, multilevel regression models were used to estimate the *a*, *b*, and *c*′ effects in order to adjust the standard errors for the presence of any intra-class correlation among students attending the same school. Models were adjusted for age, gender, race/ethnicity, and the baseline measure of the psychosocial mediator. The main outcome, initiation of sexual intercourse, was analyzed using a multilevel logistic regression model; therefore all estimates reported have been standardized in order to carry out the Sobel test [20].

## 3. Results

Table 1 shows the alpha reliability statistics for the psychosocial scales tested in the mediation models; Table 2 provides descriptive statistics on the baseline characteristics of the sample.  Table 3 provides the estimated standardized *a*, *b*, and *c*′ effects and corresponding standard errors from the multilevel regression models. 

The *a* path provides an estimate of the strength of the relationship between each psychosocial mediator and the intervention. Refusal self-efficacy, perceived norms about condoms, and STI signs and symptoms were all significantly impacted by the intervention (*P* < 0.05). While not statistically significant at the 0.05 level, perceived friend's behavior (*P* = 0.06), condom knowledge (*P* = 0.09), and HIV/STI knowledge (*P* = 0.07) were also most likely influenced by the intervention. The *b* path provides an estimate of the relationship of the proposed psychosocial mediator to the behavior outcome (initiation of sex). Of the 13 psychosocial mediators under investigation, all but 2 (perceived norms about condoms and HIV/STI knowledge) were significantly related to the behavior (initiation of sex).

The IYG study found a significant difference in the proportion of students initiating sex when compared to the control condition [7]. By the final followup, 27.4% of the study participants who reported not being sexually experienced at baseline had initiated sex. The final path, *c*′, estimates how much of this significant intervention effect was direct and not transmitted through any of the measured psychosocial mediators. This direct effect would indicate total mediation if it was estimated at or near zero when the psychosocial mediator was introduced into the model. However, the direct intervention effect remains significant for the mediator sexual beliefs (*P* = 0.048), but for the other twelve outcomes the intervention effect is no longer significant suggesting some degree of partial mediation. The *ab* path provides an estimate of the indirect effect or measure of the amount of mediation. While the proportion of intervention effect mediated by the measured psychosocial variables (indirect effect/total effect) ranged from <1% to 14.1%, only refusal self-efficacy (*P* = 0.03) and STI signs and symptoms (*P* = 0.001) were significant mediators of the intervention effect according to the Sobel test. An increase in the refusal self-efficacy scale was associated with a reduction in the likelihood of initiation of sex. However, an increase in knowledge of STI signs and symptoms was associated with an increase in initiation. These two mediators were estimated to account for 6.7 and 3.6 percent of the total intervention effect, respectively.

## 4. Discussion

The findings from this study provide evidence that the majority of the psychosocial mediators targeted by the IYG intervention are indeed related to the desired behavior and provide evidence that the conceptual theory underlying the targeted psychosocial mediators in the intervention is appropriate. The relationships between the psychosocial mediators and the behavioral outcome, the *b effects*, indicate that eleven of the thirteen mediators are related to *initiation of sex*. This provides the strongest evidence that the mediators currently being targeted by the intervention do have the potential to influence the outcome behaviors. Only two of the psychosocial mediators, *HIV/STI knowledge* and *perceived norms about condoms*, were not significantly related to the behavior.Glassman et al. (in submission)found similar *b* path results in their mediation analysis of a similar theory-based HIV/STI pregnancy prevention program, further supporting the theoretical underpinnings of these types of interventions.

While the *b* effect was in the anticipated direction for most of the mediators, two, *STI signs and symptoms knowledge* and *condom knowledge*, had an unexpected result. The results suggest that, as knowledge increases, a student has a higher likelihood of initiation of sex. One possible explanation for this inverse result is a reversal of the causal pathway, meaning that students who already are sexually active may have more knowledge about condoms and STIs. While the theoretical model assumes that the change in the mediator precedes the behavioral change, the timing of the measures in this particular study did not allow us to confirm this temporal relationship. Further analyses using a time lag should be carried out to explore this result. 

The *a* effect, which estimates the intervention's impact on the psychosocial mediators, indicates that the intervention significantly impacted three of the thirteen psychosocial variables (*STI signs and symptoms*, *refusal self-efficacy*, and *perceived norms about condoms*). While largely consistent, it should be noted that these results of the intervention's impact on the psychosocial mediators differ somewhat from those of the main outcome paper. This is most likely due to the fact that the analyses for this study were carried out on only a subset (student's who were not sexually active at baseline) of the study cohort and thus have less statistical power. For a detailed description of the intervention's impact on the psychosocial outcomes for the entire cohort, see the main outcome paper [7]. 

The program's impact on the behavior adjusting for each mediator, *c*′, was in the expected direction. All *c*′ effects indicate, there is still a reduction in sexual debut in the intervention arm relative to the control condition after adjusting for each psychosocial mediator. However, only two of the psychosocial mediators significantly mediated the intervention effect, *Knowledge of STI signs and symptoms*, and *refusal self-efficacy*. This demonstrates the importance of refusal self-efficacy skills training for this age group to delay sexual initiation. Multiple behavioral science theories (e.g., social cognitive theory, theory of planned behavior, integrative model of behavioral prediction) propose that self-efficacy is an important construct leading to behavior change. Thus, including activities that enhance students' self-efficacy to refuse to engage in unhealthy behaviors (e.g., to refuse to have sex when they do not want to) is a key strategy in many effective sexual education programs. Many middle school youth find it hard to use effective refusal skills (e.g., to say “no” clearly using a firm tone of voice, stiff body language, repeating the no message, leaving the situation), so they benefit from activities like role plays that let them practice these skills in a safe environment and to get feedback from the instructor and peers.

 Neither of the two significant mediators explained a high proportion of the intervention effect. This, combined with the lack of significance of mediating effects for the majority of psychosocial variables examined, indicates that the intervention's impact on behavior is likely influenced by mediators not measured in this study.Glassman et al. (submission pending)found similar results. The only other study found to investigate mediators of a school-based HIV/STI prevention program was conducted for middle school youth in Tanzania; they also identified only one significant mediator (peer norms regarding having sex), accounting for 16% of the total intervention effect stigler [21]. As this study also indicated, additional work needs to be conducted to help identify and better understand the mediating variables in order to better target and refine HIV/STI/pregnancy prevention interventions. In particular, results of mediation analysis suggest areas for deepening instruction (i.e., refusal self-efficacy) and perhaps bringing in additional theories to augment those areas currently covered in the intervention curriculum. 

This mediation analysis is only the first step to understanding all aspects of the complex process of behavior change resulting from a theory-based prevention intervention such as IYG [22]. These same types of analyses need to be carried out for multiple interventions across various populations to begin to fully understand where and among what populations particular aspects of the intervention should be tailored to ultimately improve the magnitude of risk reduction. Additionally, more complex multilevel mediation analysis methods, such as growth process mediation analyses [23] and multilevel structural equation modeling (MSEM; [24]) analyses that allow for decomposition of mediating effects into individual student and group (school or classroom) levels, are logical next steps in the research exploring different aspects of how psychosocial factors influence the effects of HIV/STI/pregnancy prevention programs on sexual risk-taking outcomes. 

Results of this study should not be viewed in isolation, rather taken as a piece of a larger puzzle to aid in the overall understanding of how to tailor and augment current HIV/STI pregnancy prevention programs that have already proven to be successful. Through targeting and refining these interventions for specific populations, the end goal is to achieve the largest, cost-effective risk reduction that is feasible within the confines of a school-based curriculum. 

This study is not without limitations. Outcome measures were self-report, and it is unknown the ability to generalize beyond the study population. Additionally, some of the mediators had low internal consistency. More work needs to be done in the area of measurement around these potential mediators. Authors in [21] also noted some low measurement reliability, which could have been responsible for lack of mediating effects. The study was conducted among students from 7th to 9th grade when the prevalence of sexual activity is still low, thus adversely impacting the power of the study. School-based studies such as this one often fail to reach the highest risk youth who often drop out early or have a high degree of mobility, making it difficult to track and retain them in a study over time. 

This study did not conduct mediation analyses across subgroup populations (e.g., age, gender, ethnicity). These population characteristics were controlled for in the overall analyses; however, separate models carried out within each of these groups could provide useful and cost-effective information when tailoring an intervention to a specific population. Although costly, a much larger-scale study needs to be conducted to both improve the ability to generalize as well as understand the mediating pathways and how they differ across subpopulations. 

More accurate methods for testing mediation and building confidence intervals (CIs) can be obtained using the actual distribution of the product or bootstrap methods [13, 25]; however, our models using these methods failed to converge. For this reason, the described method of inference about indirect effect estimates was used. More work needs to be done in the area of estimating these alternate methods of testing mediation effects. 

## 5. Conclusion

Results of this study suggest specific areas for deepening the focus of these types of HIV/STI/pregnancy prevention interventions for this type of middle school population. In addition, the relatively small proportion of intervention effect mediated by each of the psychosocial measures is suggestive that additional mediators not measured in this study may also be driving the intervention's effect on behavior change. Augmenting existing theories may help bolster intervention effects. Additional mediation analyses are needed that examine different aspects of the underlying mechanisms of interventions for different populations. Different stories are beginning to emerge about mediators for similar theory-based interventions when implemented in different populations (e.g.,Glassman et al., submission pending). As the body of literature in this field begins to grow, interventionists will be better equipped to begin to understand these complex processes and how to tailor intervention curriculum through targeted instruction for different populations to gain maximum risk reduction.

## Figures and Tables

**Table 1:** Psychosocial mediators.

Construct	Example item	No. of items	Response format	Alpha
*Behavioral knowledge*				
STI signs and symptoms knowledge	Symptoms of an STD, pain or burning when urinating (going to the bathroom)	5	True, false	0.46
HIV/STI knowledge	Some STDs put you at higher risk of getting infected with HIV	4	True, false, not sure	0.47
Condom knowledge	Do condoms reduce the risk of HIV, the virus that causes AIDS?	3	Yes, no, not sure	0.51

*Perceived self-efficacy*				
Refusal self-efficacy	Could you stop them if they wanted to touch your private parts below the waist?	7	4 points (definitely could not to definitely could)	0.87
Condom self-efficacy	If you wanted to get a condom, how sure are you that you could go to the store and buy one?	5	4 points (definitely could not to definitely could)	0.61

*Behavioral and normative beliefs*				
Sexual beliefs	I believe it's OK for people my age to have sex with a steady boyfriend or girlfriend	4	4 points (strongly disagree to strongly agree)	0.78
Beliefs about abstinence	I believe having sex before marriage is wrong	3	4 points (strongly disagree to strongly agree)	0.72
Perceived friends' beliefs	Most of my friends believe people should wait until they are older before they have sex	3	4 points (strongly disagree to strongly agree)	0.75
Perceived friends' sexual behavior	How many of your friends have had oral sex?	4	None, few, some, most	0.77
Perceived norms about condoms	Most of my friends believe condoms should always be used if a person my age has sex.	3	4 points (strongly disagree to strongly agree)	0.84
Reasons for having sex^+^	To feel more accepted and loved	8	[checked, not checked]	0.61
Reasons for not having sex^+^	Because I do not want a baby right now	9	[checked, not checked]	0.79

*Risky situations*				
Exposure to risky situations	In the past 3 months, how often have you been alone with someone you are very attracted to?	7	Never to 6 or more times	0.79

^+^Score represents the total number of items endorsed.

**Table 2:** Study descriptives of analysis sample at baseline.

Measure	Overall (*n* = 817)	Intervention (*n* = 308)	Control (*n* = 509)
	Mean (SE) or %	Mean (SE) or %	Mean (SE) or %
Demographics			
Mean age	12.4 (0.59)	12.5 (0.61)	12.4 (0.58)
% female	62.4%	64.9%	60.9%
African American	39.7%	43.5%	37.3%
Hispanic	46.6%	46.8%	46.6%
Other	13.7%	9.7%	16.1%
Psychosocial mediators			
Sexual beliefs	3.19 (0.63)	3.15 (0.61)	3.22 (0.63)
Beliefs about abstinence	2.88 (0.71)	2.86 (0.73)	2.90 (0.70)
Perceived friends' beliefs about sex	2.64 (0.73)	2.57 (0.78)	2.68 (0.70)
Perceived friends' behavior	1.19 (0.66)	1.20 (0.68)	1.19 (0.65)
Refusal self-efficacy (to have sex or engage in precoital behaviors)	3.19 (0.75)	3.17 (0.76)	3.20 (0.74)
Condom knowledge	1.63 (1.02)	1.69 (1.01)	1.59 (1.03)
Perceived norms about condoms	3.24 (0.69)	3.21 (0.72)	3.26 (0.67)
Condom self-efficacy	2.27 (0.43)	2.28 (0.43)	2.26 (0.43)
Exposure to risky situations	0.44 (0.50)	0.44 (0.52)	0.44 (0.48)
HIV/STI knowledge	0.56 (0.30)	0.57 (0.31)	0.55 (0.30)
Reasons for not having sex^+^	4.85 (2.58)	4.58 (2.66)	5.01 (2.52)

^+^Score represents the total number of items endorsed.

**Table 3:** Mediation of effects of intervention on initiation of sex.

	Program's effect on the			Mediator's effect on			Program's effect on the outcome			Estimate of mediated			
	mediator			the outcome			adjusting for the mediator			effect (Sobel test)			Proportion of effect
	*a* ^s^	(SE)	*P* value	*b* ^s^	(SE)	*P* value	*c* ^′s^	(SE)	*P* value	*ab* ^s^	(SE)	*P* value	that is mediated
*Behavioral knowledge*													
STI signs and symptoms^∗∗^	0.03	0.01	0.000	0.19	0.04	0.000	−0.14	0.08	0.068	0.005	0.002	0.001	3.6%
HIV/STI knowledge	0.01	0.01	0.069	−0.01	0.03	0.753	−0.11	0.07	0.093	0.000	0.000	0.757	0.1%
Condom knowledge	0.04	0.03	0.087	0.05	0.02	0.032	−0.11	0.07	0.115	0.002	0.002	0.181	1.9%

*Perceived self efficacy*													
Refusal self efficacy^∗^	0.02	0.01	0.028	−0.30	0.04	0.000	−0.10	0.07	0.188	−0.007	0.003	0.034	6.7%
Condom self efficacy	0.01	0.01	0.296	0.26	0.06	0.000	−0.12	0.06	0.061	0.002	0.002	0.311	1.7%

*Behavioral and normative beliefs*													
Sexual beliefs	0.01	0.01	0.579	−0.22	0.04	0.000	−0.12	0.06	0.048	−0.002	0.003	0.581	1.5%
Beliefs about abstinence	0.03	0.02	0.230	−0.35	0.07	0.000	−0.10	0.05	0.066	−0.010	0.008	0.243	9.3%
Perceived friends' beliefs	0.02	0.02	0.227	−0.32	0.05	0.000	−0.11	0.06	0.072	−0.007	0.006	0.237	6.3%
Perceived friends' sexual behavior	−0.03	0.02	0.059	0.50	0.06	0.000	−0.09	0.05	0.089	−0.015	0.008	0.064	14.1%
Perceived norms about condoms	0.03	0.01	0.000	−0.01	0.05	0.776	−0.11	0.07	0.119	0.000	0.002	0.776	0.4%
Reasons for having sex	0.01	0.02	0.657	0.19	0.04	0.000	−0.11	0.08	0.143	0.002	0.005	0.658	1.8%
Reasons for not having sex	0.07	0.05	0.232	−0.20	0.06	0.000	−0.10	0.06	0.098	−0.013	0.012	0.258	11.4%

*Risky situations*													
Exposure to risky situations	−0.03	0.02	0.189	0.53	0.03	0.000	−0.09	0.06	0.146	−0.013	0.010	0.191	13.3%

^s^Regression path coefficients have been standardized.

^∗^Significantly mediated intervention effect *P* < 0.05.

^∗∗^Significantly mediated intervention effect *P* < 0.01.
